# Comparative genotyping and phenotyping of *Aspergillus fumigatus* isolates from humans, dogs and the environment

**DOI:** 10.1186/s12866-018-1244-2

**Published:** 2018-09-17

**Authors:** Ivan D. Valdes, Joris van den Berg, Annika Haagsman, Natalia Escobar, Jacques F. Meis, Ferry Hagen, Pieter Jan Haas, Jos Houbraken, Han A. B. Wösten, Hans de Cock

**Affiliations:** 1 Microbiology, Department of Biology, Utrecht University, Utrecht, The Netherlands; 2 Veterinary Medicine, Utrecht University, Utrecht, The Netherlands; 3 Department of Medical Microbiology and Infectious Diseases, Canisius-Wilhelmina Hospital, Nijmegen, The Netherlands; 4 Centre of Expertise in Mycology Radboudumc/CWZ, Nijmegen, The Netherlands; 5 Westerdijk Institute, Utrecht, The Netherlands; 6 University Medical Center Utrecht, Utrecht, The Netherlands

## Abstract

**Background:**

*Aspergillus fumigatus* is a ubiquitous saprotrophic fungus and an opportunistic pathogen of humans and animals. Humans and animals can inhale hundreds of *A. fumigatus* spores daily. Normally this is harmless for humans, but in case of immunodeficiency, invasive pulmonary aspergillosis (IPA) can develop with a high mortality rate. *A. fumigatus* also causes non-invasive mycoses like sino-nasal aspergillosis (SNA) in dogs.

**Results:**

In this study we compared *A. fumigatus* isolates from humans with suspected IPA, dogs with SNA, and a set of environmental isolates. Phylogenetic inference based on calmodulin (*CaM*) and beta-tubulin (*benA*) sequences did not reveal *A. fumigatus* sub-groups linked to the origin of the isolates. Genotyping and microsatellite analysis showed that each dog was infected by one *A. fumigatus* genotype, whereas human patients had mixed infections. Azole resistance was determined by antifungal susceptibility testing and sequencing of the *cyp51A* gene. A total of 12 out of 29 human isolates and 1 out of 27 environmental isolates were azole resistant. Of the azole resistant strains, 11 human isolates showed TR_34_/L98H (*n* = 6) or TR46/Y121F/T289A (*n* = 5). Phenotypically, isolates from dogs were more variable in growth speed and morphology when compared to those isolated from human and the environment.

**Conclusions:**

1. *A. fumigatus* from dogs with SNA are phenotypically very diverse in contrast to their environmental and human counterparts.
2. Phenotypic variability can be induced during the chronic infection process in the sinus of the dogs. The basis of this heterogeneity might be due to genomic differences and/or epigenetic variations.
3. Differences in dogs is a could be a result of within-host adaption and might be triggered by environmental factors in the sinus, however this hypothesis still needs to be tested.

**Electronic supplementary material:**

The online version of this article (10.1186/s12866-018-1244-2) contains supplementary material, which is available to authorized users.

## Background

*Aspergillus fumigatus* is a filamentous saprotrophic ascomycete that occurs world-wide [1]. This fungus produces large numbers of conidia that are released into the air [2–5]. Estimates indicate that humans can inhale a few hundred of these asexual spores of *A. fumigatus* a day [2, 6]. Their small diameter of 2–3 μm and hydrophobic surface enables them to reach the alveoli [7]. Yet, most of them are trapped and removed by the mucociliary escalator or by immune cells present in the lung tissue. Upon germination, they can cause invasive or non-invasive forms of aspergillosis. The former is characterised by the penetration and infection of tissues [8]. *A. fumigatus* has been shown to secrete mycotoxins like gliotoxin, verruculogen, fumagillin and helvolic acid, which can affect ciliary beat frequency and promote colonization of lung epithelium [9, 10]. Invasive aspergillosis most often occurs in individuals with impaired cell-mediated immunity [8]. Additionally this fungus is able to cause mycosis in animals such as cats and dogs [11]. For instance, it is responsible for most cases of sino-nasal aspergillosis in dogs (SNA) [12]. Canine SNA is clinicopathologically similar to chronic erosive non-invasive fungal sinusitis in humans [13]. It is characterized by the formation of fungal plaques (i.e. fungal biofilms) in the nasal cavity and frontal sinus. Other than in some invasive forms of aspergillosis in humans [14], no evidence of impaired innate or adaptive immune responses have been described for dogs suffering from SNA [15, 16]. Notably, mesocephalic and dolichocephalic dogs seem to be affected with a higher frequency as compared to brachycephalic dogs for reasons that remain elusive but which could be related to a smaller sino-nasal surface area of the latter [13]. In cats *A. fumigatus* is one of the causative agents of upper respiratory aspergillosis (URTA). URTA in cats can be divided into sino-nasal aspergillosis (SNA) and sino-orbital aspergillosis (SOA) of which SOA is the most common condition. SNA in cats resembles SNA in dogs and is caused primarily by *A. fumigatus* but also by *Aspergillus niger*. SOA, on the other hand, is most commonly caused by *Aspergillus felis* sensu lato [17]. Still, these infections in humans, dogs and cats are, like most fungal infection in mammals, relatively uncommon, especially considering the ubiquity of *A. fumigatus* conidia in the environment.

Fungal infections are treated with a range of antimycotic compounds. The antifungal activity of azoles is based on the binding to ergosterol synthase. Both *cyp51A* and *cyp51B* of *A. fumigatus* encode for this lanosterol 14α-demethylase. Azole resistance induced mutations have been described mainly in *cyp51A* [18–20]. These mutations can involve tandem repeats in the promotor area in combination with point mutations in the cyp51A gene. Of these, the TR_34_/L98H [21, 22] and TR_46_/Y121F/T289A [23, 24] are most frequently reported with an incidence of up to 20% % in the Netherlands depending on the site of isolation of the resistant strain [25]. Additionally it has been suggested that about 10% of azole resistant strains that does not possess a mutation of the *cyp51A* gene can be explained by increased activity in ABC transporters that helps to detoxificate the fungal cell from azoles, or mutation in other genes like *hapE* [20, 26, 27].

Here we performed a comparative phenotypic and genetic analysis of *A. fumigatus* isolates from dogs with SNA, human patients suspected of having invasive *A. fumigatus* infection from a well-defined group of high-risk patients from the haematology ward and the intensive care unit (ICU), and isolates from indoor and outdoor substrates. Phylogenetic inference based on calmodulin (*CaM*) and beta-tubulin (*benA*) sequences did not reveal *A. fumigatus* sub-groups but dog isolates were more variable in growth speed and morphology. Possibly, *A. fumigatus* evolves within the dog during the infection process by mutations and / or epigenetic variations.

## Methods

### Fungal isolates and culturing

A total of 87 isolates of *A. fumigatus* were used (Table 1; Additional file 1: Table S1) that were derived from the environment (*n* = 27), infected dogs (*n* = 27), and humans (*n* = 29). *A. fumigatus* var. *ellipticus* type-strain [28], *A. fumigatus* Af293, and *A. fumigatus* Af1163 were included as reference strains. *A. fumigatus* isolates were originated from 9 dogs (Canine patient CP1–8.2, Additional file 1: Table S1) suffering from SNA presented at the Faculty of Veterinary Medicine in Utrecht between 2010 and 2012. One dog (CP8) was presented twice at the hospital due to a reinfection after 2 years (CP8.2 with isolates DTO 303-F1 and -F2, Additional file 1: Table S1). Hospitalized canine patients suspected to suffer from SNA were subjected to computer tomography (CT), and endoscopic/rhinoscopic, and haematological analysis. Clinical signs specific of SNA are unilateral or bilateral mucopurulent nasal discharge, depigmentation of the nasal planum, an increased airflow through the nostrils, and an enlarged ipsilateral mandibular lymph node. In the case a fungal plaque was detected (Additional file 1: Table S1), tissue and blood samples were sent to the Veterinary Pathology Diagnostic Center and the Veterinary Diagnostic Laboratory of Utrecht University. Haematocrit, leucocytes, blood cell differentiation, total protein, albumin and the protein spectrum was measured in the blood samples.

**Table 1:** Overview of *A. fumigatus* isolates

	Number of isolates	Provided by	Country of origin	Reference/Year
Environmental	23	Westerdijk Institute Utrecht	The Netherlands and Germany	2005–2008
	4	Westerdijk Institute Utrecht	Ireland	[4], 2005
Canine isolates	27	Clinical Sciences of Companion Animals, Veterinary Medicine, Utrecht University	The Netherlands	2010–2012
Human isolates	29	University Medical Centre Utrecht	The Netherlands	[30], 2011–2013
*Aspergillius fumigatus* Af293 (CBS 126847)	1	Westerdijk Institute Utrecht	UK	[51], 1993
*Aspergillius fumigatus* A1163	1	Westerdijk institute Utrecht	France	[52], 2008
*Aspergillius fumigatus* var*. ellipticus*	1	Westerdijk Institute Utrecht	USA	[28], 1965

Fungal plaques were obtained via trephination of the frontal sinus and/or rhinoscopic removal in the nasal sinus with a 30 degrees 2.7 mm rigid endoscope and using a combination of suction and a metal hook. Trephination was performed if mycotic plaques in the frontal sinus were diagnosed by CT. After removal of the fungal plaques the nasal sinus was flushed in most cases with clotrimazol for 15 min each in ventral, left and right lateral, and dorsal recumbency, followed by flushing for 15 min in ventral recumbency, after which the clotrimazol was allowed to leave the nasal sinus by gravity (head down). Part of the fungal plaque was immediately frozen in liquid nitrogen and stored at − 80 °C. Remaining material was placed on ice and used for microbial culturing and histological analysis. Culturing was started at the same day on potato dextrose agar (PDA, Becton Dickinson, Le-Pont-De-Claix, France) and care was taken to inoculate plates with different pieces of fungal plaques of the same dog. Colonies incubated for 2 days at 37 °C presenting macroscopical characteristics of *A. fumigatus* were sub-cultured on PDA plates using vegetative mycelium from the outer part of the colony as an inoculum. Conidial suspensions were made as described [29].

Isolates from *A. fumigatus* were obtained from 9 high-risk patients from the intensive care and haematology units of the Utrecht University Medical Center (UMCU) who were suspected to have developed invasive aspergillosis (patients HP1–9; Additional file 1: Table S1) but none of them had received prophylactic antifungal therapy. These isolates were obtained between 2011 and 2013 from sputum, bronchoalveolar lavage, or pleural fluid (Additional file 1: Table S1; [30]). The human isolates were provided as colonies on Malt Extract agar (MEA) plates. In order to eliminate the possibility of having more than one isolate per plate, colonies were sub-cultured on PDA plates using vegetative mycelium from the edge and the centre of the colonies as inoculum. Plates were inoculated by tracing a double zigzag pattern with a plastic inoculating loop. After incubation at 37 °C for 3 days, plates were inspected for the presence of colonies with different morphology. Clearly distinct colonies were treated as different isolates. Conidial suspensions were prepared as described [29].

### Phenotypic characterization of fungal isolates

Phenotypic characterization was performed on creatine sucrose agar (CREA), Czapek yeast agar (CYA), CYA with 5% NaCl (CYAS), dichloran 18% glycerol agar (DG18), malt extract agar (MEA), yeast extract sucrose agar (YES) as recommended [29]. A 3-point inoculation was used and plates were incubated lid-side up at 25 °C for a period of 7 days in the dark. Diameter of colonies was analysed by ImageJ [31] after taken photographs from a fixed height with a Canon camera with a telephoto lens. They were subjected to PCA and k-means clustering with k = 3 corresponding to the initial number of groups (i.e. indoor and outdoor substrates, dog, and human). In addition, colour of the medium underneath the colony, spatial distribution of conidia formation, and morphology of the conidiophores was analysed. Pictures of conidiophores and conidia were made as described [29]. Mycelium was scraped of colonies that had been grown on MEA for 7 days. This mycelium was mounted on an object glass in a drop of lactic acid and a very small drop of ethanol (70%) was used to flush the excess conidia outward. Samples were studied using Olympus BH2 and Zeiss Axioskop microscopes.

### DNA isolation, sequencing, genetic and phylogenetic analysis

Genomic DNA was isolated with the UltraClean Microbial DNA Isolation kit (MO BIO Laboratories, Solana Beach CA) and used to PCR amplify fragments of calmodulin (*CaM*), β-tubulin (*benA*), and the mating type loci *MAT1–1* and *MAT1–2* (Table 2; [29]). Sequencing of the reverse and forward strands was performed using the Big Dye® Terminator Cycle Sequencing Ready Reaction kit (Applied Biosystems, Foster City, CA, USA). Products of the sequencing reactions were purified using Sephadex G-50 gel filtration (GE Healthcare, Little Chalfont, Buckinghamshire, UK), equilibrated in double-distilled water, and analyzed using the ABI PRISM 310 Genetic Analyzer (Applied Biosystems). The *CaM* and *benA* sequences of the isolates and *A. niger* NRRL326, *A. clavatus* NRRL1, *A. felis* CBS130245, *A. lentulus* NRRL35552, *A. fumigatus* NRRL5109, *A. fumigatus* NRRL164, *A. fumigatus* NRRL163, and *N. fischeri* NRRL181 were used to construct a phylogenetic inference. Phylogenetic trees were constructed using the ETE Toolkit pipeline [32] and visualized with Figtree v1.4.2 (http://tree.bio.ed.ac.uk/software/figtree/).

**Table 2:** Primers and temperature conditions used for PCR [29]

Locus	Primer	Annealing temp (°C)	Cycles	Direction	Sequence (5′-3′)	Reference
*CaM*	CMD5	55	35	Forward	CCGAGTACAAGGARGCCTTC	[53]
	CMD6	55	35	Reverse	CCGATRGAGGTCATRACG TGG	
*BenA*	Bt_2_a	55	35	Forward	GGTAACCAAATCGGTGCTGTTC	[54]
	Bt_2_b	55	35	Reverse	ACCCTCAGTGTAGTGACCCTTGGC	
*MAT1–1*	AFM1_F65655 MAT1_R6215	48 48	30 30	Forward Reverse	CCTYGACGMGATGGGITGG TGTCAAAGARTCCAAAAGGAGG	this study
*MAT1–2*	MAT2_F6086 MAT2_R6580	48 48	30 30	Forward Reverse	TCGACAAGATCAAAWCYCGTC CTTYTTGARCTCTTCYGCTAGG	this study

### Microsatellite analysis

Microsatellite genotyping was performed using the STR*Af* assay as described [33]. All nine markers data were imported into R package poppr [34] to construct a minimum spanning tree. Bruvo distance was used to determine genetic diversity between isolates [35].

### TR profiling and microdilution assay

TR_34_ and TR_46_ repeat analysis within *cyp51A* was performed using qPCR as described [36], additional information about the sequence of the cyp51A was available for human isolates (Additional file 1: Table S1). Microdilution assays to determine Minimum inhibitory concentrations (MIC) of itraconazole (Santa Cruz Biotech, Dallas, USA), posaconazole (MSD, Kenilworth, USA) and voriconazole (Pfizer, New York, USA) were performed according to EUCAST (http://www.eucast.org/fileadmin/src/media/PDFs/EUCAST_files/AFST/Files/EUCAST_E_Def_9_3_Mould_testing_definitive.pdf).

## Results

### Histology and haematological analysis

CT scan and rhinoscopy confirmed sinonasal mycosis in 9 dogs presented at the Veterinary hospital at Utrecht University. Histopathological biopsies showed non-invading hyphae on the mucosal surface. The lamina propria was not invaded (Additional file 2: Figure S1). Conidiophores were rarily observed, which is consistent with the observation that fungal plaques in the nasal cavities were white (Additional file 2: Figure S1B). The lamina propria was infiltrated by large amounts of lymphocytes, plasma cells, and neutrophil granulocytes. Haematological analysis showed differences in the levels of blood albumin in all dogs. Four dogs showed signs of mild blood loss in the weeks before the treatment, which could explain the hypoalbuminemia. Together, all 9 dogs suffered from a chronic lymphoplasmacellular rhinitis with fungal hyphae and ulcerations.

### Genotyping and phylogeny

A phylogenetic tree of the *A. fumigatus* isolates was constructed using the *benA* and *CaM* genes (Additional file 3: Figure S2). In 4 out of 87 isolates only one of these genes was amplified successfully (Additional file 1: Table S1). The tree based on *CaM* (Additional file 3: Figure S2) showed no subgroups of isolates. They all grouped with *A. fumigatus* Af293 and NRRL 163 (Additional file 3: Figure S2, left) but not with the emerging fungal pathogen *A. felis* [17] (Additional file 3: Figure S2). The same result was obtained when the tree was constructed with *benA* sequences (Additional file 3: Figure S2, right), indicating that these markers are unable to distinguish between the set of isolates used in this study.

**Fig. 1:**
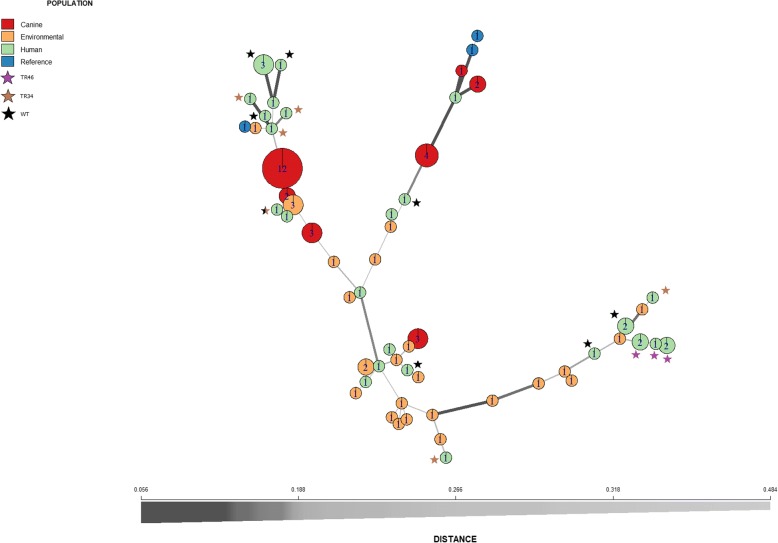
Minimum spanning tree of *A. fumigatus* isolates. Circles indicate different genotypes, numbers inside the circles indicate the number of isolates belonging to that particular genotype, thickness of the line indicates the relatedness of the connected isolates determined by Bruvo distance; values close to zero indicate identical isolates, while values close to 1 indicate unrelated isolates [35]. Additionally, TR mutations present in the isolates are depicted as color-coded stars. The black star (WT) refers to azole sensitive phenotype

**Fig. 2:**
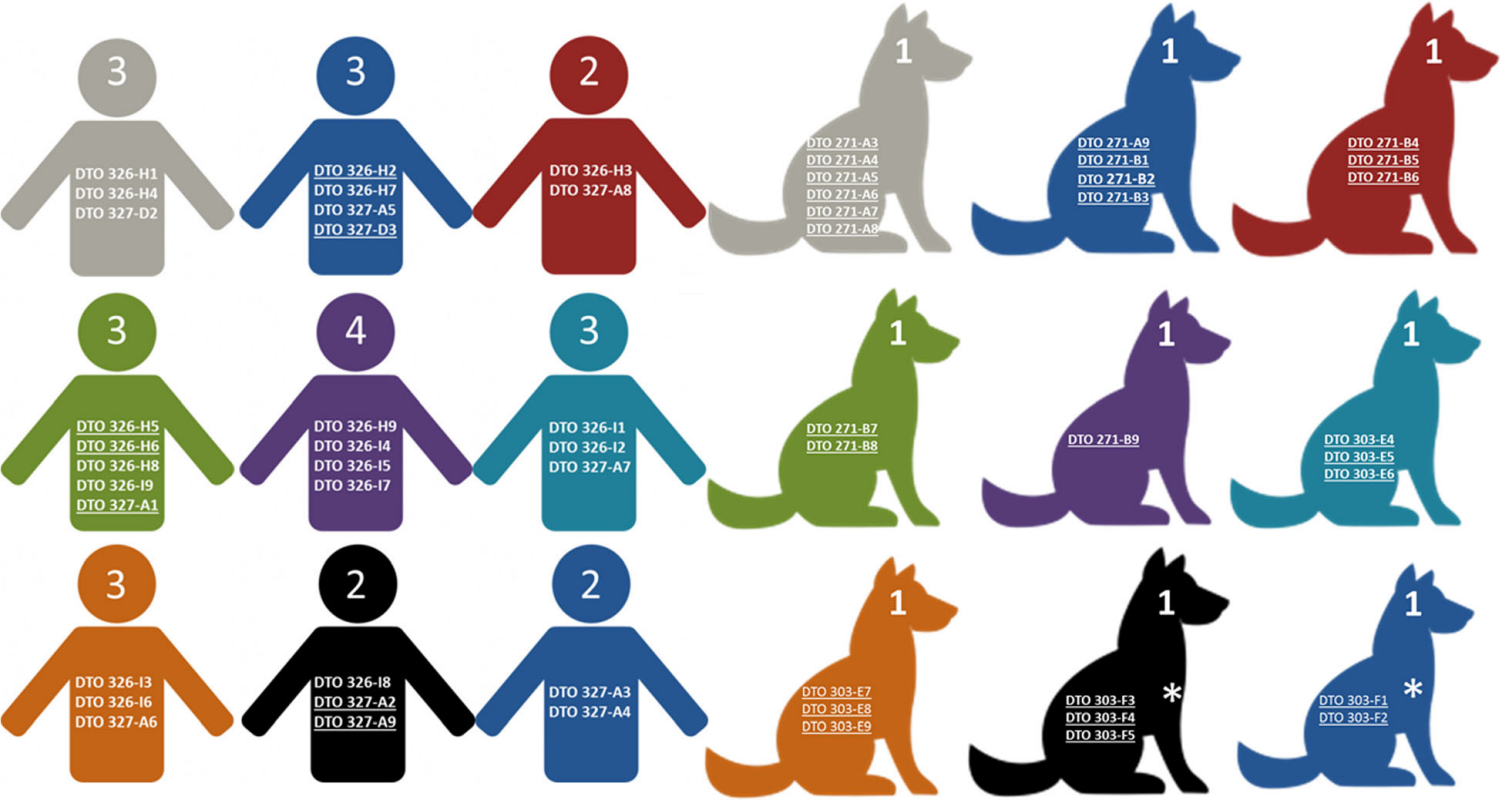
Schematic overview of the nine human and canine patients. The total number of genotypes and the codes of the *A. fumigatus* isolates from each patient is indicated. Isolates that are underlined belong to the same genotype in the case of canine patients, an asterisk indicates the same patient (CP8 – CP8.2) being re-infected with a different *A. fumigatus* isolate

Fungal isolates from an individual dog were of the same mating type, while both mating types were present in individual human patients (4 out of 9). The 9 infected dogs had either isolates with *MAT1.1* (4) or *MAT1.2* (5). The fungal isolates from humans (15 isolates having *MAT1.1* and 12 isolates having *MAT1.2*) and indoor and outdoor substrates (12 isolates having *MAT1.1* and 13 isolates having *MAT1.2*) also showed a ratio close to 1. Because of the inability of the previous markers to differentiate between isolates, genetic heterogeneity was further analyzed by microsatellite analysis using the STR*Af* assay. A total of 59 *A. fumigatus* genotypes were found within the 87 isolates with a genetic distance between the isolates ranging between 0.056 and 0.496 (Fig. 1). Most of the isolates were found to be of a unique distant genotype. In the case of indoor and outdoor substrates only two genotypes were represented by more than one isolate. Similarly, most of the isolates from a human individual were of a different genotype (Fig. 2). Moreover, genotypes were different between human individuals except for genotype 10 (Additional file 1: Table S1) which was found in two human patients. In contrast, the 27 dog isolates from the 9 dogs grouped only in six clusters. The isolates derived from an individual dog all had the same genotype (Fig. 2, Additional file 1: Table S1).

### Phenotyping of *A. fumigatus* isolates

**Fig. 3:**
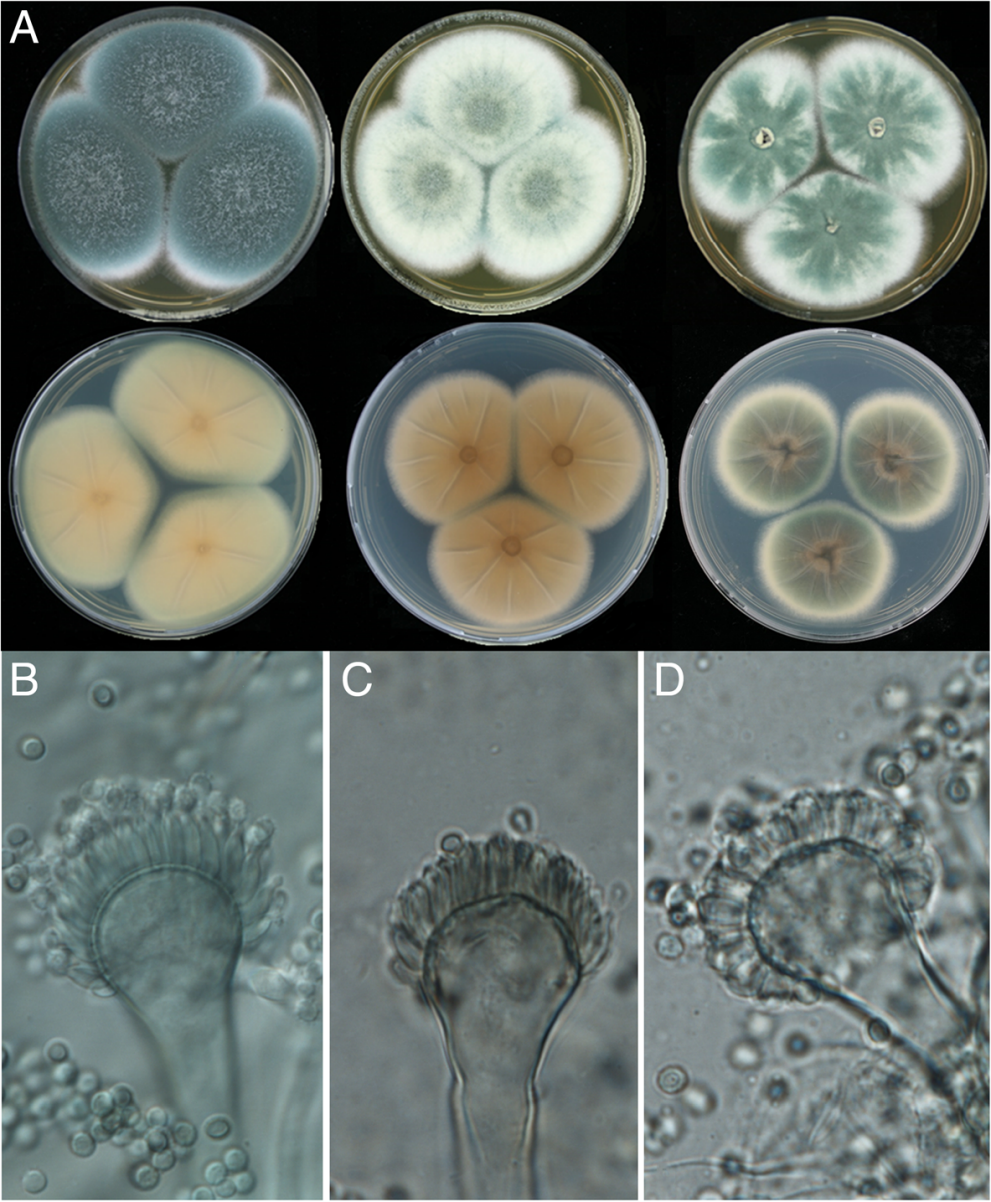
Morphology of colonies (**a**) and conidiophores and conidia (B-D) of *A. fumigatus* isolates from dogs, humans and the environment. Top row of panel A shows the obverse side of the colony grown on MEA of isolates DTO 028-D6, DTO 271-A5, and DTO 303-E8, while the bottom row shows the reverse side of CYA-grown colonies of DTO 028-D6, DTO 326-I9, DTO 326-H2. A conidiophore of DTO 327-D3 with flask-shaped phialides and abundant production of conidia is shown in (**b**), while (**c**) shows similar phialides of isolate DTO 326-I9 with sparse production of conidia. Reduced, cylindrical shaped phialides of DTO 303-F2 and abundant production of conidia are shown in (**d**)

**Fig. 4:**
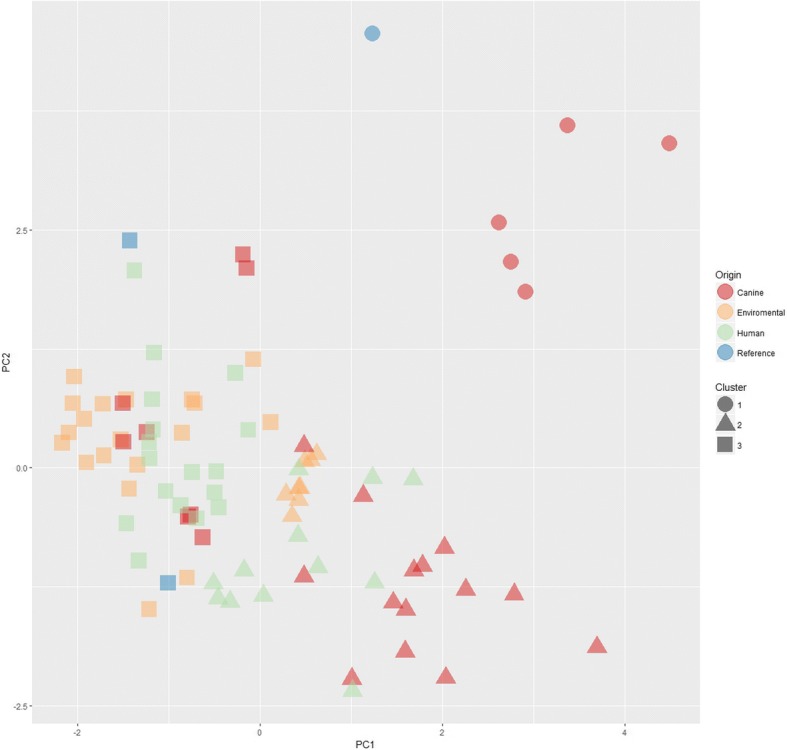
PCA and K-means clustering of *A. fumigatus* isolates based on colony grown on several media. The arrow indicates the position of *A. fumigatus* var. *ellipticus*

Most isolates showed a colony and conidiophore morphology typical for *A. fumigatus*(Fig. 3). However, the isolates from dogs were phenotypically more diverse as compared to those from the environment or humans (Tables 3 and 4). Variation was also observed between isolates from a single dog. This was the case for dogs CP1, CP2, CP4, CP7 and CP8.2. Conidiophore morphology was atypical in 12 out of 27 isolates from dogs. Furthermore, whitish colonies with concentric green rings of conidia forming conidiophores were observed in 12 out of the 27 fungal isolates (Fig. 3). This phenotype was associated with reduced production of conidia as compared to the fungal isolates from human patients and outdoor en indoor substrates. Acidification of CREA medium was not observed in the case of human isolates and the reference strains but was detected in 7 out of 27 fungal isolates from dogs and 12 out of 27 environmental isolates (Table 3 and Additional file 1: Table S1). Additionally, high variation was observed in the average diameter of colonies of isolates from dogs grown on CREA, MEA, YES, DG18, CYAS (Table 5). PCA and k-means clustering using colony diameter as a proxy for growth showed that one cluster contained only 5 isolates from dogs (DTO 303-E6, DTO 303-F5, DTO 303-E5, DTO 271-B3, DTO 271-B2) as well as *A. fumigatus* var. *ellipticus* (cluster 1 in Fig. 4) The isolates from indoor and outdoor substrates and human patients were distributed in clusters 2 and 3. Fourteen and eight isolates from dogs clustered in clusters 2 and 3, respectively (Fig. 4).

**Table 3:** Number of isolates from human, dog and indoor and outdoor substrates per phenotypic trait

	Phenotype	Canine (27)	Environmental (27)	Human (29)	Reference (3)
Degree of sporulation and phialide shape in MEA	Strong sporulation and cylindrical phialides	1			
	Strong sporulation and flask-shaped phialides	15	27	29	3
	Sparse sporulation and flask-shaped phialides	11			
	Dark greenish colony	14	27	29	2
Sporulation in MEA	Mostly whitish colony, with the center of the colony green followed by concentric rings of pale yellowish green	12			1
	Whitish colony with star-like green patches	1			
	Acid production absent	20	15	29	3
Acid production on CREA	Acid production present	7	12		
	Brownish	1		3	
Reverse color on CYA	Green with brown center and white margins		1	2	1
	White	26	26	24	2

Total number of isolates per source is indicated between brackets

**Table 4:** Distribution of phenotypes of *A. fumigatus* isolated from 9 dogs suffering from SNA

	Phenotype	CP1 (6)	CP2(4)	CP3(3)	CP4 (2)	CP5(1)	CP6(3)	CP7(3)	CP8(3)	CP8.2(2)
Degree of sporulation and phialide shape in MEA	Strong sporulation and cylindrical phialides									1
	Strong sporulation and flask-shaped phialides	1	1	3	2	1	3	3		1
	Sparse sporulation and flask-shaped phialides	5	3						3	
Sporulation in MEA	Dark greenish colony	1	1	3	1	1	3	2		2
	Mostly whitish colony, with the centre of the colony green followed by concentric rings of pale yellowish green	5	3		1				3	
	Whitish colony with star-like green patches							1		
Pigmentation in CREA	No pigment	5	4		2	1		3	3	2
	Yellow pigment	1		3			3			
Pigmentation in CYA	Brownish reverse		1							
	White reverse	6	3	3	2	1	3	3	3	2

The total number of isolates per dog is between brackets

**Table 5:** Mean diameter with standard deviation of colonies (mm) on each medium for each investigated isolate set

Medium	Dog isolates	Environmental isolates	Human isolates	References
CREA	31.81 ± 10.52	26.73 ± 6.90	29.55 ± 7.98	25.31 ± 12.02
CYA	36.90 ± 11.25	44.57 ± 4.62	42.44 ± 4.32	39.98 ± 10.96
CYAS	28.66 ± 10.76	25.54 ± 3.53	24.83 ± 6.81	14.74 ± 11.30
DG18	28.65 ± 9.99	19.92 ± 2.85	28.67 ± 8.76	11.56 ± 11.86
MEA	36.37 ± 11.04	44.55 ± 5.96	45.63 ± 3.87	39.06 ± 11.22
YES	38.98 ± 11.90	49.02 ± 3.20	47.36 ± 5.72	41.78 ± 13.26

### TR profiling and resistance to azoles

Microdilution assays showed that 12 out of the 29 human isolates were resistant to itraconazole, posaconazole and/or voriconazole. Of these isolates, 6 showed a TR_34_ promotor duplication in *cyp51A* and 5 a TR_46_ duplication in this gene. One resistant (DTO 060-G9) and 3 intermediate resistant (DTO 028-D6, DTO 086-C1, DTO 089-G1) posaconazole strains were identified in the environmental isolates from the Netherlands. Dog isolates were all sensitive to the azoles tested, and both groups of strains (Dog and environmental) did not show TR_34_ and TR_46_ mutations. The Af293 strain was sensitive to itraconazole and posaconazole and resistant to voriconazole like the results presented by Mowat et al. [37] (Additional file 1: Table S1) and the EUCAST MIC distribution for Af293 (Additional file 4: Figure S3) Af293 is however generally considered susceptible for this azole.

## Discussion

We assessed phenotypic and genetic differences between strains of *A. fumigatus* isolated from indoor and outdoor substrates and from infected humans and dogs. Genetic analyses did not indicate the existence of sub-groups within *A. fumigatus* specialized in infecting human or dog. A notable variation in phialide morphology, sporulation, colony size, and colony color was observed between strains isolated from dogs suffering from SNA; even between isolates from the same infected individual. This phenotypic variability was not associated with genetic differences in the *benA, CaM, MAT1–1, MAT1–2, cyp51A* loci and microsatellites. This indicates that the dogs were infected with a single strain. It is tempting to speculate that the phenotypic differences evolve within the dog during the infection process due to epigenetic changes or mutations. The fact that phenotypic variability was hardly observed in isolates from human and from indoor and outdoor substrates suggests that the dog sinus induces phenotypic evolution. SNA in dogs is a chronic infection starting with an inoculum in the front of the sinus [13]. This may be the cause of the observed variation in the dog isolates. A long exposure to a stressful environment and for example host immune response, nutrient, and gas (oxygen) availability may act as a selection pressure [36]. Reduced asexual reproduction, as observed in 11 out of 27 dog isolates can be an adaptation to increase fitness in the host [38, 39]. Similarly, higher growth rate at 37 °C have been correlated with increased virulence [40]. Notably, in our study isolates from dog and humans had a higher growth rate on DG18 media as compared to the environmental isolates. The real meaning of this variation of growth for virulence is unclear and requires further investigation.

The observation that a single genotype persist in a dog could suggest that the spread of the fungus in the sinus proceeds gradually associated with a non-sporulating biofilm but not invading the epithelium of the sinus [41]. The infection within human patients is also different from dogs because human patients can carry multiple *A. fumigatus* strains [42–44]. This was confirmed in our study by the presence of *MAT1–1* and *MAT1–2* strains isolated from an individual patient that had different microsatellite profiles. It should be noted that it is difficult to associate the human isolates with a proven infection since they were obtained via bronchoalveolar lavage or sputum samples and not from deep tissue samples.

Clinical azole resistance in the Netherlands is 5–20% for *A. fumigatus* isolated from human patients [25, 30, 45]. The majority (89%) contain TR_34_/L98H or TR_46_/Y121F/T289A mutations, while 11% of the resistant isolates carry wild type *cyp51A* [46]. In our study, tandem repeats TR_34_ or TR_46_ were only detected in human clinical isolates. A single posaconazole resistant isolate from environmental isolates was found which was not TR associated and future genome sequencing should illuminate the resistance mechanism involved. Jensen and colleagues [47] found few azole resistant environmental isolates (4/133) in the National Mycology Reference Laboratory of Denmark. In contrast, Klaassen et al. [48] reported that 16 out of the 213 clinical isolates and 9 of the 42 environmental isolates contained the TR_34_/L98H allele associated with multi-triazole-resistance. Clearly, extensive azole fungicide use in the environment can result in selection of azole resistant strains and complicate clinical management [49]. A survey including whole genome sequencing (WGS) between environmental and clinical isolates can give clues about the evolution of resistance in both niches [50].

## Conclusion

Taken together our results showed that phenotypic and genetic heterogenicity is a key factor to understand several aspects of infections caused by *A.fumigatus*. Dog SNA isolates were phenotypically more diverse than human and environmental isolates. Additionally, using the STRAf assay we observed that fungal plaques from dog suffering SNA harbour isolates genetically identical (one genotype), while humans with suspected IPA are infected with multiple genotypes. Phenotypic variation in dog isolates might be due to genomic differences or epigenetic variations and due to host adaption during the chronic infection process in dogs. New experiments aiming to understand the genomic, epigenetic and physiological adaptations of the isolates studied can provide clues host-driven evolution and its implication in virulence and control of the disease caused by this pathogen in humans.

## Additional file

Additional file 1: **Table S1.** Excel file with detailed phenotypic and genetic data .n.d, not determined (no PCR product or sequence obtained) (XLSX 20 kb)

Additional file 2: **Figure S1.** Histology section of the mucosa of a dog with SNA (A) and rhinoscopic image of the fungal plaque within the sinus nasalis (B). Arrow in (A) indicates mucosa tissue with high infiltration of immune cells. Note that the hyphae indicated with a star in (B) do not penetrate the epithelial tissue (TIF 2505 kb)

Additional file 3: **Figure S2.** Phylogenetic inference constructed using *CaM* (left) and *benA* (right) sequences of human, dog, and indoor and outdoor isolates as well as reference strains. Green represents indoor and outdoor isolates, blue and red represent isolates from dogs and human, respectively, and black represents reference strains. *A.felis* indicated with *. (TIF 2808 kb)

Additional file 4: **Figure S3.** Voriconazole MIC distribution for Microdilution assay from EUCAST (consulted 22/06/2018), note that MIC of 4 mg/L are reported. (JPG 108 kb)

## Abbreviations

CREA: Creatine sucrose agar
CT: Computed tomography scan
CYA: Czapek yeast agar
CYAS: Czapek yeast agar + 5% NaCl
DG18: Dichloran 18% glycerol agar
EUCAST: European Committee on Antimicrobial Susceptibility Testing
IPA: Invasive pulmonary aspergillosis
MEA: Malt extract agar
MIC: Minimum inhibitory concentration
PCA: Principal components analysis
PDA: Potato dextrose agar
SNA: Sino nasal aspergilosis
SOA: Sino orbital aspergillosis
STR*Af*: Short tandem repeats assay for *Aspergillus fumigatus*
