# Supplementary figures and images for: Comparative genotyping and phenotyping of Aspergillus fumigatus isolates from humans, dogs and the environment
Source: BMC Microbiol. 2018 Sep 17;18:118. doi: 10.1186/s12866-018-1244-2 (PMC6142626; doi:10.1186/s12866-018-1244-2)

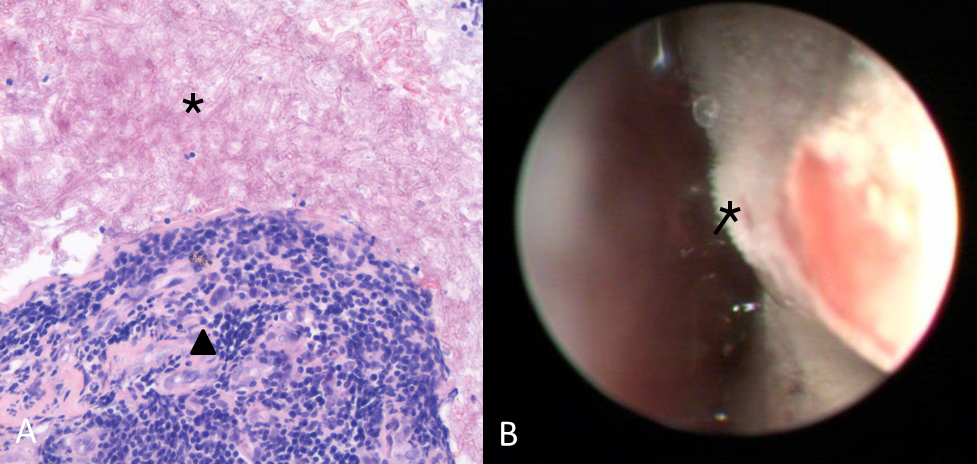

Supplement: Supplementary file 2 — Figure S1. Histology section of the mucosa of a dog with SNA (A) and rhinoscopic image of the fungal plaque within the sinus nasalis (B). Arrow in (A) indicates mucosa tissue with high infiltration of immune cells. Note that the hyphae indicated with a star in (B) do not penetrate the epithelial tissue (TIF 2505 kb) [file 12866_2018_1244_MOESM2_ESM.tif]

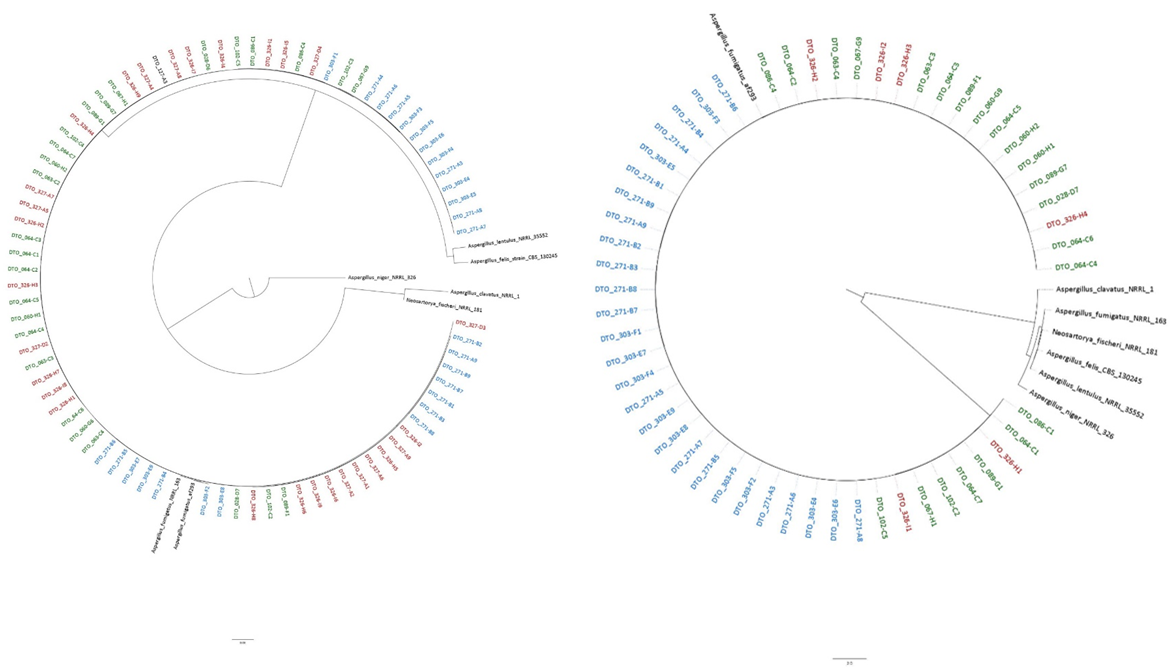

Supplement: Supplementary file 3 — Figure S2. Phylogenetic inference constructed using CaM (left) and benA (right) sequences of human, dog, and indoor and outdoor isolates as well as reference strains. Green represents indoor and outdoor isolates, blue and red represent isolates from dogs and human, respectively, and black represents reference strains. A.felis indicated with *. (TIF 2808 kb) [file 12866_2018_1244_MOESM3_ESM.tif]

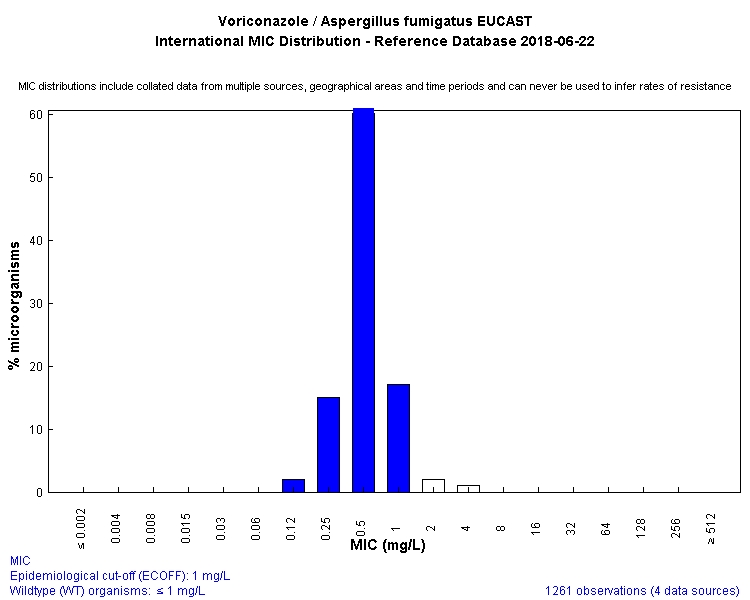

Supplement: Supplementary file 4 — Figure S3. Voriconazole MIC distribution for Microdilution assay from EUCAST (consulted 22/06/2018), note that MIC of 4 mg/L are reported. (JPG 108 kb) [file 12866_2018_1244_MOESM4_ESM.jpg]
